# Maltraitance scolaire et maladie de Basedow: à propos d’un cas

**DOI:** 10.11604/pamj.2017.26.15.11317

**Published:** 2017-01-12

**Authors:** Marie-Pierrette Ntyonga-Pono, Daniela Nsame

**Affiliations:** 1 Faculté de Médecine de Libreville et Service d’Endocrinologie du Centre Hospitalier Universitaire de Libreville, Libreville, Gabon; 2 Service d’Endocrinologie du Centre Hospitalier Universitaire de Libreville, Libreville, Gabon

**Keywords:** Graves-Basedow’s disease, child, student abuse, Graves-basedow's disease, child, student abuse

## Abstract

La maladie de Basedow, thyréopathie auto-immune, est la cause la plus fréquente des hyperthyroïdies aussi bien chez l'adulte que chez l'enfant. Elle relèverait d'une prédisposition génétique sur laquelle vont intervenir des facteurs déclenchants environnementaux dont le stress. Le but de cet article est de présenter un cas de maladie de Basedow chez l'enfant dont le facteur déclenchant inhabituel serait la maltraitance scolaire, vaste problème aux conséquences multiples.

## Abstract (en)

Graves-basedow's disease is an autoimmune thyreopathy which represents the most common cause of hyperthyroidism in both adults and children. This disease would be related to a genetic predisposition influenced by environmental triggers, including stress. This study aims to present a case of Graves' disease in children whose unusual trigger would be student abuse, a broad challenge with multiple implications.

## Introduction

La maladie de Basedow ou de Graves, maladie auto-immune de la glande thyroïde, est la cause la plus fréquente des hyperthyroïdies qui touchent 0,5 à 2% de la population adulte [1]. Chez l'enfant par contre, la maladie de Basedow est une affection plutôt rare, mais sa prévalence augmente dans le monde, notamment en Asie [2]. Les causes exactes de ce désordre immunitaire ne sont pas bien connues mais on évoque le rôle de différents facteurs environnementaux déclenchant, dont le stress, sur un terrain génétiquement prédisposé [3, 4]. Le but de ce travail est de rapporter un cas de maladie de Basedow chez un enfant dont le facteur déclenchant serait la maltraitance scolaire, ce qui permet de discuter du rôle du stress dans le déclenchement de cette pathologie.

## Patient et observation

Il s'agit d'un garçon de 7ans, premier d'une fratrie de quatre enfants, scolarisé en première année primaire qui nous a été adressé de Lambaréné où il vit avec ses parents, pour prise en charge d'un amaigrissement important avec exophtalmie évoluant depuis 10 mois. Il est né au terme d'une grossesse suivie régulièrement. L'interrogatoire n'a pas retrouvé d'antécédents personnels ni familiaux pathologiques particuliers notamment pas de notion de maladie auto immune. La famille n'a pas de problèmes particuliers, le père est monogame. Les symptômes remonteraient à environ 10 mois par la constatation d'un amaigrissement contrastant avec une polyphagie et une énurésie avec refus de se rendre à l'école. Les parents ne comprenaient pas ce qui arrivait à l'enfant et ont consulté plusieurs médecins dans leur ville d'origine sans succès jusqu'au dernier médecin qui a pensé à un problème thyroïdien et nous l'a adressé. En interrogeant directement l'enfant sur ce qui lui était arrivé au début de sa maladie, il nous a raconté dans ses propres mots que le « maître lui avait damé un coup de chicotte sur la tête parce qu'il avait eu tout faux » L'examen clinique a objectivé : Un enfant amaigri pesant 21kgs pour 142cm, dyspnéique avec une labilité émotionnelle pleurant facilement. Il présentait un goitre visible de loin et une exophtalmie. Le goitre était diffus, soufflant à l'auscultation avec un frémissement palpatoire traduisant son hyper vascularisation. Il n'y avait pas d'adénopathies jugulo-carotidiennes satellites. L'examen cardiovasculaire mettait en évidence : une turgescence spontanée des veines jugulaires, un bruit de galop et un souffle d'insuffisance mitrale coté 3/6. La fréquence cardiaque était à 130 par minute, la pression artérielle à 120/68 mmHg. L'examen pleuro-pulmonaire était sans particularité. L'examen neurologique objectivait un tremblement fin des extrémités, une hyperactivité motrice. L'examen cutanéo-muqueux mettait en évidence de multiples lésions de grattage secondaires à un prurit généralisé. L'examen de l'appareil génito-urinaire était sans particularité. L'enfant a été hospitalisé dans le service d'endocrinologie du centre hospitalier universitaire de Libreville où des examens complémentaires ont été demandés. Ce bilan à montré les résultats suivants : TSHus<0,05µUI/ml (normale 0,4-4,8), T4 Libre>100 pmol/l (normale 9-25). Échographie thyroïdienne : glande thyroïde en situation anatomique, globalement augmentée de volume, sans nodule individualisable ni calcifications. ECG : tachycardie sinusale à 135/min, hypertrophie ventriculaire gauche (HVG) avec indice de Sokolov=60mm. Hémogramme : Globules blancs =10860/mm^3^ dont 6% de polynucléaires neutrophiles, 61% de lymphocytes, 7,5% d'éosinophiles soit 810/mm^3^; Hémoglobine=11,6g/dl avec un volume gobulaire moyen = 74,2 fl (anémie microcytaire); plaquettes =383000/mm^3^. Fonctions rénale et hépatique normales. Glycémie veineuse : 3,5mmol/l. Le traitement prescrit associait: Carbimazole (Neomercazole*) à la dose de 0,5mg/kg/j, Propanolol (Avlocardyl*) 0,5mg/kg/j et un anxiolytique ainsi qu'un déparasitage systématique à l'albendazole à cause de l'hyperéosinophilie. Sous ce traitement l'évolution a été rapidement favorable, malgré le fait que cet enfant présentait une maladie de Basedow plutôt sévère avec une cardiothyréose [5]. Il est actuellement au traitement d'entretien pour 18 mois qui devrait se terminer en Janvier 2018 mais malheureusement les parents ne respectent pas les dates de contrôle.

## Discussion

L'hyperthyroïdie chez l'enfant est plus rare que chez l'adulte la cause la plus fréquente étant la maladie de Basedow dont l'incidence croît avec l'âge, rare avant 5 ans mais avec un pic d'incidence entre 9 et 15 ans [2, 6]. Cette incidence varie selon les régions, plus élevée chez les Asiatiques que chez les Caucasiens, pouvant atteindre 14.1/100.000/année à Hong Kong [7]. Les facteurs déclenchant cette thyréopathie auto-immune ne sont pas bien connus [3]. On évoque une prédisposition génétique sur laquelle différents facteurs comme le stress, le tabagisme, l'apport d'iode dans les régions carencées, comme ce qui a dû probablement se passer pour quelques un des cas rapportés au Mali [6], certains médicaments, l'accouchement, vont déclencher la réaction auto-immune [8, 9]. Cette réaction va se traduire par la production d'anticorps anti récepteurs de la TSH qui vont stimuler la croissance des cellules folliculaires et augmenter la production d'hormones thyroïdiennes triiodothyronine (T3) et tétraiodothyronine (T4) [9]. Parmi les facteurs déclenchants, le stress jouerait un rôle particulier en perturbant le système endocrinien et les hormones ainsi produites en excès, notamment les gluco-corticoides, perturberaient le système immunitaire [3]. Les évènements stressants de la vie les plus cités sont la perte d'un conjoint, la perte d'un travail, les difficultés financières, les conflits avec le partenaire, les conflits avec un supérieur, la prise en charge d'un proche malade, et les situations traumatisantes de la vie dont les mauvais traitements [8, 9]. Chez l'enfant, une forte composante familiale serait retrouvée dans le déclenchement de la maladie [10]. Ce n'est probablement pas le cas de notre jeune patient qui vit dans une famille monogamique sans problèmes. Nous rattachons sa maladie plutôt au traumatisme vécu à l'école qui a eu des répercussions profondes sur son psychisme comme le traduit l'apparition d'une énurésie secondaire [11]. La maltraitance scolaire physique est encore hélas une réalité dans notre système éducatif mais elle se retrouve aussi sous d'autres formes même dans les pays développés comme le montrent ces témoignages d'enfants et de parents dont les enfants ont été des victimes [12]. Cette maltraitance scolaire entre dans le cadre plus large de la maltraitance des enfants, vaste problème mondial selon l'OMS qui peut avoir de graves répercussions sur la santé physique et mentale des victimes [13]. Enfin on peut aussi discuter du problème thérapeutique à long terme posé par cet enfant. En effet le suivi irrégulier laisse présager de problèmes à long terme car il est rapporté que les rechutes sont pus fréquentes chez l'enfant et le traitement médical doit être poursuivi plus longtemps, jusqu'à 5 ans au lieu de 18 mois [2]. C'est ainsi que certains auteurs proposent un traitement radical [2].

## Conclusion

La maladie de Basedow est la cause la plus fréquente des hyperthyroïdies aussi bien chez l'adulte que chez l'enfant. Les causes de cette pathologie auto-immune sont multiples, génétiques et environnementales et chez l'enfant on devrait prêter attention non seulement aux problèmes familiaux mais aussi au vécu scolaire comme l'illustre cette observation.
